# Extrachromosomal circular DNA in cancer drug resistance and its potential clinical implications

**DOI:** 10.3389/fonc.2022.1092705

**Published:** 2023-01-30

**Authors:** Juanjuan Luo, Ying Li, Tangxuan Zhang, Tianhan Xv, Chao Chen, Mengting Li, Qixiang Qiu, Yusheng Song, Shaogui Wan

**Affiliations:** ^1^ Center for Molecular Pathology, Department of Basic Medicine, Gannan Medical University, Ganzhou, China; ^2^ China Medical University, Shenyang, China, Ganzhou, China; ^3^ Department of Interventional Radiology, The People’s Hospital of Ganzhou City, Ganzhou, China

**Keywords:** cancer genetics, extrachromosomal circular DNA, drug resistance, chromothripsis, genomic instability

## Abstract

Chemotherapy is widely used to treat patients with cancer. However, resistance to chemotherapeutic drugs remains a major clinical concern. The mechanisms of cancer drug resistance are extremely complex and involve such factors such as genomic instability, DNA repair, and chromothripsis. A recently emerging area of interest is extrachromosomal circular DNA (eccDNA), which forms owing to genomic instability and chromothripsis. eccDNA exists widely in physiologically healthy individuals but also arises during tumorigenesis and/or treatment as a drug resistance mechanism. In this review, we summarize the recent progress in research regarding the role of eccDNA in the development of cancer drug resistance as well as the mechanisms thereof. Furthermore, we discuss the clinical applications of eccDNA and propose some novel strategies for characterizing drug-resistant biomarkers and developing potential targeted cancer therapies.

## 1 Introduction

Cancer is the leading cause of death in China, and chemotherapy is one of the main treatments for cancer patients (1, 2). However, cancer patients frequently develop resistance to chemotherapy during treatment. Drug resistance as one of the main reasons for clinical chemotherapy failure is defined as the decline of drug effects during treatment (3). It can be categorized as intrinsic resistance and acquired resistance (4). Intrinsic resistance predates treatment and refers to the ability of a population of cells within a treatment-naive cancer to survive initial therapy due to a preexisting genetic alteration or cell state, whereas acquired resistance develops by the acquisition of new mutations, metabolic adaptations, and epigenetic changes in the original cancer (5). The drug resistance in most cancer cells belongs to acquired drug resistance, which is gradually produced in the process of chemotherapy. As a result, the curative effect becomes worse and most cancer patients have no available chemotherapeutics in their late-stage treatments. Therefore, it is one of the most urgent problems to be solved in treating malignant tumors. There are many mechanisms that are involved in the drug resistance of cancer cells, including genomic instability, DNA repair, chromothripsis, and drug-target mutations (3, 6, 7). These factors involve a series of genetic changes, and the formation of extrachromosomal circular DNA (eccDNA) is closely related to these genetic factors (5, 8). Recently, more and more studies have suggested that eccDNA is involved in the resistance to cancer treatment (9, 10).

eccDNA refers to a type of single-stranded or double-stranded circular DNA that originates from but is likely independent of chromosomes, and its size varies from hundreds of base pairs (bp) to several megabases (Mb) (11). According to different sizes and sequences, eccDNA is further categorized into microDNA (100–400 bp), small poly-dispersed DNA (spcDNA) (100 bp–10 kb), telomeric circles (t-circles) (multiples of 738 bp), and the largest extrachromosomal DNA (ecDNA) (millions of bp). These DNA molecules can carry oncogenic driver genes or increase the copy number of genes to regulate cancer growth and drug resistance (11). Some microDNA may be transcribed into some functional small regulatory RNAs, including microRNAs and novel small interfering RNAs, which can mediate cancer development or cancer drug resistance by regulating gene expression patterns (12, 13). The generation of spcDNA is closely related to genomic instability, which can cause genetic variation on a genome-wide scale (14). These variations will give cancer cells the advantage of clonal growth and genetic evolution and could ultimately cause tumorigenesis. The t-circle can effectively lengthen telomeres through rolling circle amplification and play an important role in alternative lengthening of telomeres (ALT), affecting cancer cell proliferation and cell-cycle progression (15, 16). The large ecDNA, also known as the double minute chromosome (DM), can directly encode some drug-resistant genes or increase the copy number of genes to regulate drug resistance. Moreover, most studies suggested that the large-size eccDNA, termed as ecDNA, is involved in drug resistance, instead of the small-size eccDNA. Therefore, in this review article, we will summarize the role of ecDNA in cancer drug resistance and its molecular mechanism. Furthermore, we also propose its clinical applications, which will provide new strategies for screening drug-resistant biomarkers to improve targeted therapy effects.

## 2 The role of eccDNA in cancer drug resistance

In recent years, with the rise of high-throughput sequencing technology, the structure and genetic characteristics of eccDNA have been gradually revealed. eccDNAs have been found in most cancers, and their role in the drug resistance of many cancers has been widely explored (17). The roles and identification methods of eccDNA in the drug-resistant process of various cancers are summarized in **Table 1** by cancer types.

**Table 1:** Summary of eccDNA in chemotherapy drug resistance by cancer types.

Cancer	Host genes	Drugs	Samples	Detection methods	Reference
Glioblastoma	EGFRvIII	Erlotinib	GBM39 cell	Single-cell analyses	(18)
Glioblastoma	MDM2	Erlotinib	GBM cell	FISH, PCR, and Southern blot	(18)
Glioblastoma	ABCG2	Mitoxantrone	SF295 cells	FISH	(19)
Neuroblastoma	MYCN	–	NB tumor biopsy tissue	FISH and qPCR	(20)
Cervical carcinoma	DHFR	Methotrexate	HeLa S3 cell	FISH and *in situ* Hi-C sequencing	(7)
Cervical carcinoma	DHFR	Methotrexate	HeLa cell	FISH	(21)
Colon cancer	DHFR	Methotrexate	HT29 cell	RT-PCR and FISH	(22)
Human epidermal carcinoma	MDR1	Vinblastine	KB-V1 cell	Electrophoresis of DNAs and gamma-irradiation	(23)
Human epidermal carcinoma	MDR1, MDR2	Colchicine	KB carcinoma cell	Southern blot, Giemsa staining, and pulsed-field gel electrophoresis	(24)
Hypopharyngeal squamous cell carcinoma	RAB3B	Cisplatin	FaDu cell	Circle-seq	(25)
Oral squamous cell carcinoma	MDR1	Vinblastine	KB cell	DNA electrophoresis and DNA probe	(26)
Small-cell lung carcinoma	DHFR	Methotrexate	NCI-H249P,NCI-H187 cell	Dot blot hybridization	(27)
Choriocarcinoma	DHFR	Methotrexate	HCCM and CC1 cell	Giemsa staining and Southern blot	(28)
Breast cancer	DHFR	Methotrexate	EMT-6 cell	Pulsed-field gel electrophoresis	(29)
chronic myelogenous leukemia	DHFR	Methotrexate	HAP1 cell	CRISPR-C; ddPCR	(30, 31)

### 2.1 The eccDNA in glioblastoma

Glioblastoma (GBM) is the most common and malignant primary brain cancer in adults with a poor prognosis and high risk of chemotherapy resistance (32). So far, the epidermal growth factor receptor (EGFR) and ATP-binding cassette subfamily G member 2 (ABCG2) have been reported frequently as drug resistance-related genes that are carried by ecDNA in GBM.

EGFR deletions and point mutations are often found in GBM, of which 50% have EGFR gene amplification in ecDNA, but 30%–60% of EGFR genes are mutated, and the most common mutation is EGFRvIII (33). EGFRvIII activates the NF-κB (nuclear factor κB) pathway and increases the aggressiveness of GBM, and cancer cells expressing EGFRvIII are more sensitive to EGFR tyrosine kinase inhibitors (TKIs) (34, 35). Nathanson et al. (18) used erlotinib to treat GBM-loading mice; 80% of the mice had a reduction in cancers, but cancer cells changed from predominantly high EGFRvIII expression to low EGFRvIII expression, accompanied by a decrease in drug sensitivity. The loss of ecDNA with EGFRvIII in erlotinib resistance was specific, as these cells still contained abundant ecDNA that carry other genes, such as murine double minute 2 (MDM2). In addition, they also found that MDM2 gene amplification was also associated with drug resistance during the study through fluorescence *in situ* hybridization (FISH) and polymerase chain reaction (PCR). After erlotinib treatment, the copy number of ecDNA with the MDM2 gene was increased and remained elevated, even after drug withdrawal.

The ABCG2 gene is located on chromosome 4, and the protein it encodes can efficiently transport a variety of chemotherapeutic drugs (36). Rao et al. (19) detected DM carrying ABCG2 gene amplification in the SF295 MX50 and MX100 sublines. They generated these sublines by exposing SF295 cells belonging to GBM to mitoxantrone. Interestingly, with the increase in mitoxantrone concentration, fewer DMs were observed, but homogeneously staining regions (HSR) that carried ABCG2 gene amplicons were visible through FISH. Obviously, amplification of ABCG2 occurred initially in the form of DM, followed by chromosomal reintegration of the amplicon at multiple sites and producing stable genotypes associated with drug resistance.

### 2.2 The eccDNA in neuroblastoma

Neuroblastoma is the most common solid extracranial neoplasm in children, showing an appreciable heterogeneity in clinical evolution. Amplification of the MYCN oncogene in this cancer is detected in 20–30% of cases and is associated with non-effective chemotherapy (37, 38). Through FISH and quantitative PCR analyses, Valent et al. (20) found that the MYCN oncogene can be amplified by ecDNA, especially in patients with advanced neuroblastoma who were resistant to chemotherapy. The MRP gene encodes special transmembrane glycoproteins, which can act as plasma membrane drug-efflux pumps, discharge drugs from the cells, eliminate the accumulation of drugs in cells, and make cancer cells acquire tolerance to varieties of drugs (39, 40). In neuroblastoma, the MYCN gene can be amplified with the help of ecDNA, which increased the expression of this gene and then upregulated the expression of multidrug resistance genes, resulting in enhanced resistance. Therefore, reducing the expression of MYCN may avoid the occurrence of chemotherapy resistance. It was reported that hydroxyurea induced the overexpression of MDR1 in cells to reduce the expression of extrachromosomal MYCN (41, 42), which provides a treatment target for the high-risk neuroblastoma in clinical settings.

### 2.3 The eccDNA in cervical carcinoma

Cervical carcinoma is the most common female reproductive system cancer in developing countries (43). Chemotherapy is considered as the standard treatment for patients with advanced or recurrent cervical cancer. Resistance to chemotherapy substantially affects the efficacy of cervical cancer treatment. Michael et al. (44) used FISH to analyze cervical cancer cells with methotrexate (MTX) resistance and found that all cells could amplify the dihydrofolate reductase (DHFR) gene *via* DMs. In addition, they found that chromosome 5 fragmentation events could form HSR with the DHFR gene. HSR could break again and produce fragments because of its instability, then produce DMs. Then, in HeLa cells, it was also confirmed that the defects in homologous recombination (HR) could play a role in the amplification of extrachromosomal DNA elements. HR-deficient cell lines had a significantly higher frequency of gene amplification, and the clone frequency of all MTX-resistant cells was higher than that of HeLa parental cells (21). Recently, Shoshani et al. (7) also performed whole-genome sequencing of clonal isolates developing MTX resistance, and the results further identified chromothripsis as a major driver of DM amplification in DMs and proved the amplification of DHFR genes in DMs, which enabled HeLa cells to rapidly acquire tolerance to altered growth conditions.

### 2.4 The eccDNA in colon cancer

Morales et al. (45) studied the resistance of colon cancer HT29 cells to MTX and the dynamic process of DHFR amplification. They characterized the DHFR genome region at the cytogenetic and molecular levels in HT29 cells treated with increasing doses of MTX. HSRs were the main form of DHFR amplification in the process of increasing the dose of MTX. DMs with the DHFR gene appear in large numbers only after the cells have been exposed to higher doses of drugs for 3 months, and cancer cell resistance to MTX also increases. HT29 cells’ resistance was reduced after withdrawal of the drug, and the sensitivity of these cells to MTX was restored. At the same time, the DM carrying the DHFR gene also disappeared. Some studies showed that the homologous recombination activity of MTX-resistant cells containing DM was increased compared with MTX-sensitive cells. With the silence of the key player BRCA1 in the HR pathway, the attenuation of HR activity decreased the number of DMs and DM-form-amplified gene copies (such as DHFR, ZFYVE16, and MSH3) and increased the exclusion of micronuclei and nuclear buds that contained DM-form amplification, which were accompanied by increased MTX sensitivity (46). Similar studies had also reported that non-homologous end joining (NHEJ) decreased MTX resistance and cell proliferation in MTX-resistant colon cancer cells, which was related to blocking of the generation of DM and the exclusion of DHFR. Therefore, the DNA repair pathway might represent a novel target to reverse drug resistance and improve therapeutic outcome by eliminating extrachromosomal amplification in cancer (22).

### 2.5 The eccDNA in human epidermal carcinoma

In KB cells from human epidermoid carcinoma, the amplified multidrug resistance (MDR) genes were contained in DM molecules; cells were then treated with colchicine to analyze MDR amplification events. As the concentration of colchicine increased, circular DNAs (890 kb) harboring MDR dimerized to large DM structures (1,780 kb) by intramolecular homologous recombination, which then dimerized to form the larger DMs (3,560 kb). Their studies revealed that the dimerization of circular amplicons was the crucial mechanism for DM generation and MDR gene amplification. Colchicine exposure also induced the mutation of the MDR1 gene. The mutated MDR gene residing on an extrachromosomal DNA element underwent random segregation at mitosis and then enhanced drug resistance to cancer cells in KB cells (24). Joseph et al. (23) also revealed that DM molecules contained the amplified MDR1 genes. Although there were few DMs in each cell, there was a >100-fold amplification of the MDR1 gene. MDR1 overexpression results in cross resistance to a variety of lipophilic compounds, including anthracene-clines (e.g., doxorubicin) and vinca alkaloids (e.g., vinblastine). In addition, Sanchez et al. (47) emphasized that fractionated ionizing radiation obviously reduced the extrachromosomal copy number of MDR1 in KB cancer cells, and this decrease was accompanied by a reduction in multidrug resistance and in P-glycoprotein levels, which might help to improve the efficacy of anticancer therapies.

### 2.6 The eccDNA in hypopharyngeal and oral squamous cell carcinoma

Hypopharyngeal squamous cell carcinoma (HSCC) was an aggressive form of head and neck squamous cell carcinoma (HNSCC) that had a poor prognosis and was rapidly rising in incidence (48). Cisplatin (DDP)-based chemotherapy was an important factor impairing the effectiveness of chemotherapy for HSCC (49, 50). Lin et al. (25) recently identified more than 10,000 eccDNA in DDP-resistant FaDu cell samples from HSCC and amplified encoding genes (such as RAB3B and RAD54L) from eccDNA (chr1^circle 46219–52682 kb^) that carried different gene fragments. Furthermore, research found that RAB3B could promote DDP resistance in hypopharyngeal squamous cell carcinoma by inducing autophagy. However, loss of MDR1-carrying ecDNA induced by hydroxyurea increased the sensitivity of vinblastine in oral squamous cell carcinoma (OSCC), and the specific mechanism by which hydroxyurea induced to accelerate loss of extrachromosomal amplified genes is still unclear, one of which may involve the formation of micronuclei. Hydroxyurea did not deeply affect the synthesis of cell DNA and preferentially inhibited the replication of DNA outside chromosomes (26). These studies suggest that eccDNA might play a significant role in cancer drug resistance by amplifying related functional genes, and we need to explore further the novel mechanisms of eccDNA in drug resistance.

### 2.7 The eccDNA in other cancers treated with MTX

In cancer cells from a patient with small-cell lung carcinoma (SCLC) who received MTX treatment, a large number of DMs were discovered, and the DHFR gene was amplified and overexpressed. During serial passages of this cell line in drug-free medium, the number of DMs and the expression level of DHFR declined. The results showed that cancer cells were more sensitive to MTX and the prevalence of DMs in metaphase cells correlated with the concentrations of resistant MTX (27). Amplification of the DHFR gene on DMs led to an increase in mRNA and protein and provided the MTX resistance of human choriocarcinoma cells (28). A similar phenomenon was also observed in mouse EMT-6 cells from breast cancer. The cells that had been irradiated and subjected to stepwise increases in MTX concentration were detected as having numerous DMs. These studies showed that ecDNA-mediated gene amplification played an important role in the MTX resistance of cancer cells. The DHFR genes on DMs were also amplified in a dose-dependent manner (29). Recent studies used CRISPR-C, which is a technology that uses Clustered Regularly Interspaced Short Palindromic Repeats to generate extrachromosomal circular DNA (30), to generate ecDNA containing the dihydrofolate reductase (DHFR) gene in the HAP1 cell line of chronic myelogenous leukemia in humans. In the absence of methotrexate, the cells maintain their initial ecDNA copies. Then, the ecDNA copy number of DHFR ecDNA rose in a strong, dose-dependent pattern in response to MTX treatment (31).

## 3 Mechanism of eccDNA driving cancer drug resistance

eccDNA can promote cancer drug resistance development in various ways, and most of these ways are related to gene amplification. It is a common manifestation of genomic instability and plays an important role in cancer progression and drug resistance (10).

### 3.1 eccDNA increases tumor heterogeneity

Cancers are not static entities: they start from a genetically normal cell and end with billions of malignant cells that have accumulated a large number of mutations in the process. Most of those occur during chromosome replication in mitosis (51). Due to the accumulation of these mutations, tumor heterogeneity is promoted, which is characteristic of malignancies (52). The existence of eccDNA is an important factor driving genetic heterogeneity in cancer. It can further enhance the heterogeneity of cancer cells, depending on its unique genetic mechanism.

**Figure 1:**
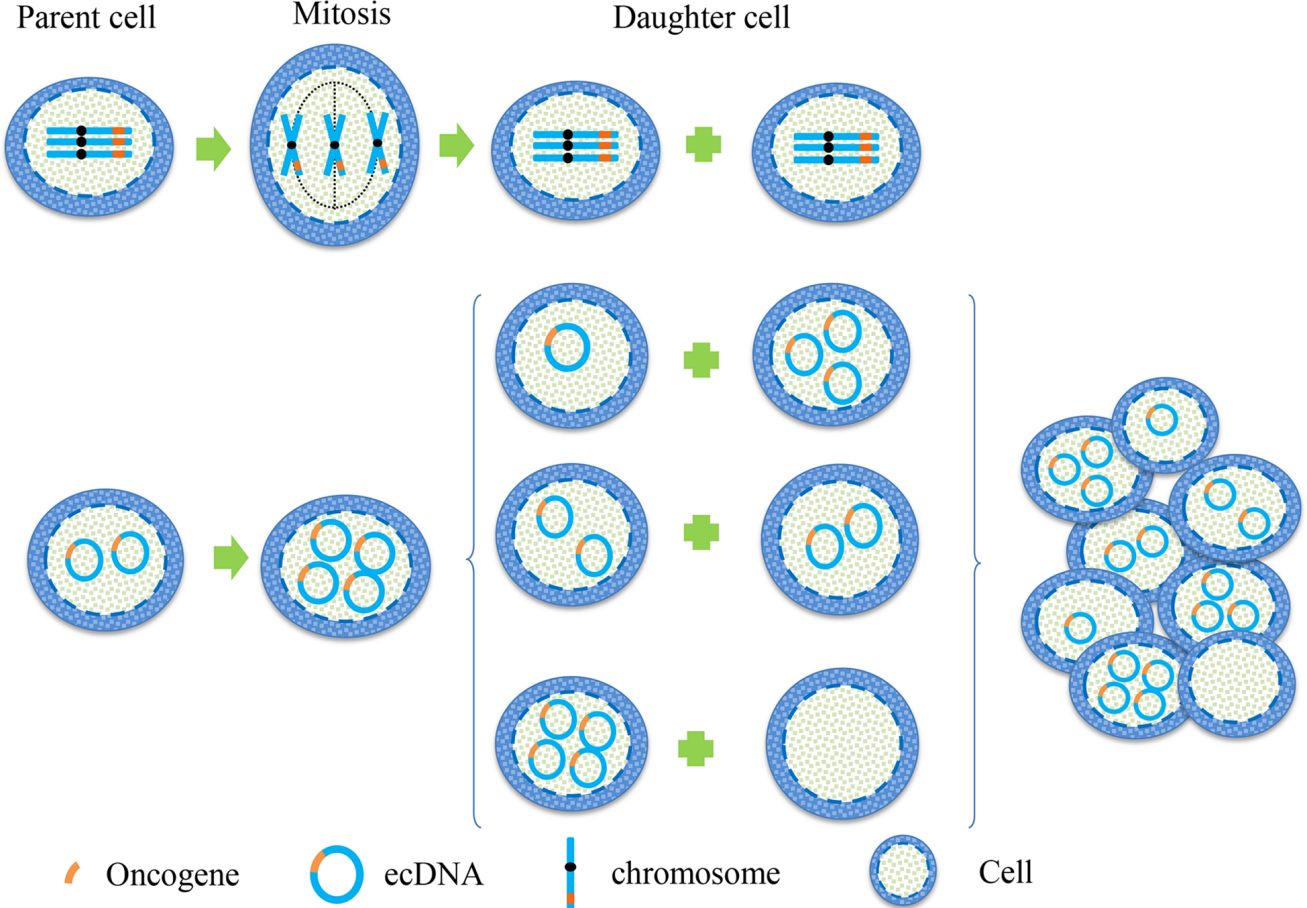
Hereditary difference between chromosome and eccDNA. When the oncogene is on a chromosome, the two sister chromatids are separated by pulling the centromere by the spindle during mitosis, and then they were equally distributed between the two daughter cells. This is a classic example of Mendel’s Law. When the oncogene is located on eccDNA, eccDNA replicates with the chromosome during mitosis. Because eccDNA has no centromere, the separation of eccDNA is not controlled by the spindle, resulting in the copied eccDNA being randomly assigned to daughter cells during cell division. This unique genetic mode makes the number of eccDNA in each cell unequal, leading to heterogeneity of eccDNA in each cell subgroup.

In cytogenetics, the inheritance of eccDNA is not the same as that of chromosomes, which does not follow Mendel’s law of inheritance (**Figure 1**). It is randomly distributed to daughter cells during mitosis, making the number of eccDNA in daughter cells unequal (17). Consequently, one of these daughter cells may have multiple oncogene copies of eccDNA during each division, thereby gaining a proliferation advantage. This approach enhances genomic diversity and promotes tumor heterogeneity, which helps cancer adapt to different environmental changes. It enables different cell subsets to have different sensitivities to therapeutic agents.

### 3.2 eccDNA is involved in gene expression associated with drug resistance

**Figure 2:**
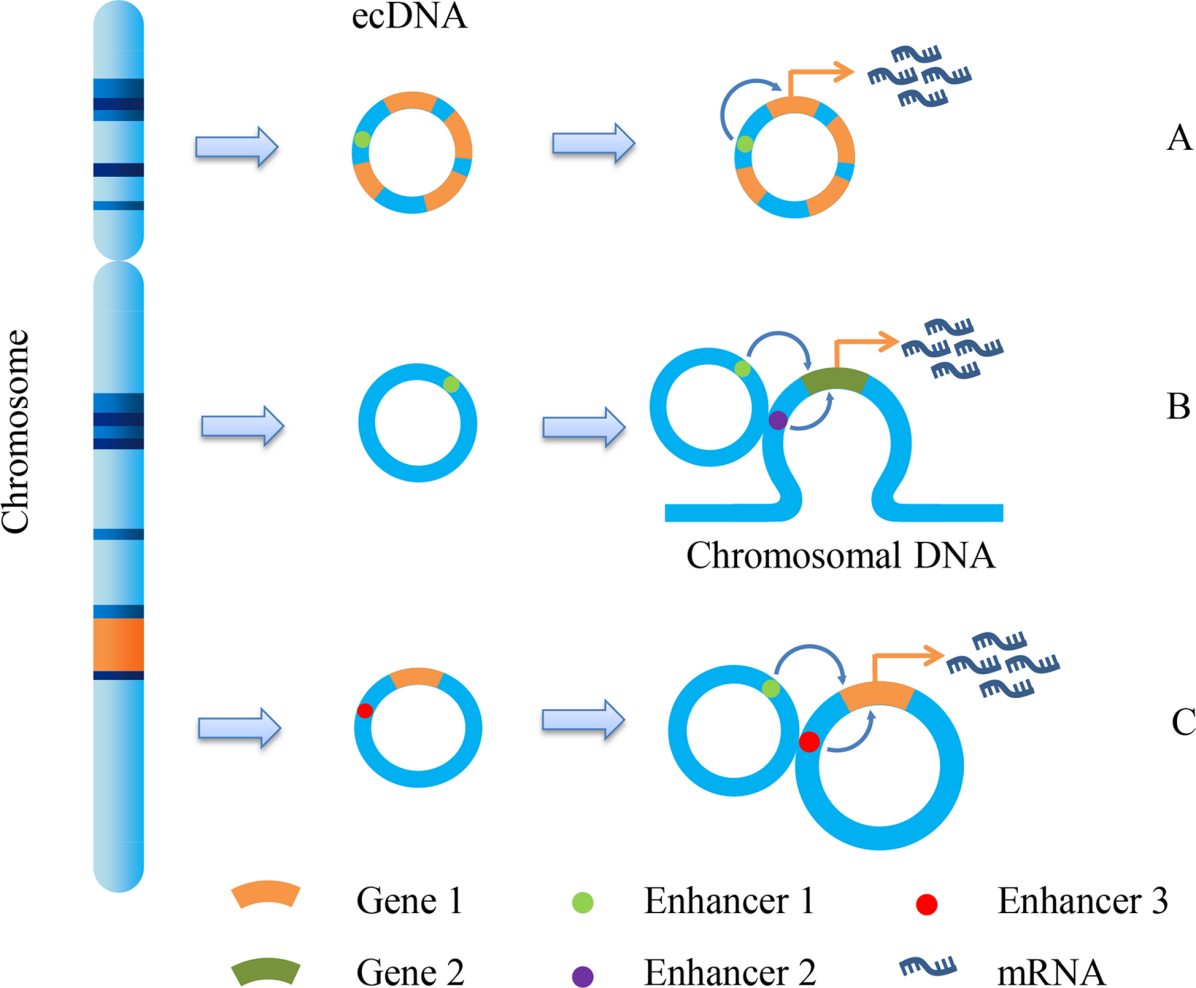
ecDNA promotes gene transcription. Due to the different fragments carried by ecDNA, it promotes gene transcription and expression in different ways. There are mainly three methods shown in the figure: **(A)** Enhancer1, located on ecDNA, can promote the expression of gene 1; **(B)** Enhancer1, located on ecDNA, can promote the expression of gene 2 on chromosomes; **(C)** the enhancer 1 on the ecDNA can promote the expression of gene 1 on another ecDNA, and the enhancer 3 on this ecDNA can also promote the expression of gene 1.

Early studies have proved that eccDNA is an important form of oncogene amplification, and its contribution to oncogene expression is mainly caused by the increase in gene copy number (10). eccDNA has the same complete domain as chromatin, although it lacks the higher-order compression state of chromosomes (53). Therefore, genes on eccDNA are more easily transcribed than those on chromosomes. The enhancers carried by eccDNA molecules can drive the transcription of their own genes and promote the transcription of other eccDNA molecules and even genomic genes (**Figure 2**) (54). We can speculate that both the increase in copy number and the high transcriptional activity of eccDNA itself can enable the overexpression of oncogenes. On the premise that ecDNA may carry a variety of genes, including oncogenes and drug resistance genes, ecDNA can make cancer cells resistant through gene amplification. Andrew et al. detected copy number alterations in 4,577 human cancer samples representing nine different solid cancers and discovered that cell-derived enhancers were co-amplified with oncogenes in multiple solid tumors, including MYCN, which bore a compact relationship with drug resistance in medulloblastoma (55). Another significant study on ecDNA in GBM revealed the molecules’ function as mobile transcriptional enhancers, which were features of widespread intra-ecDNA and genome-wide chromosomal interactions. It was co-located in the same chromatin structure region to regulate the transcriptional activity of specific genes. These genes also included MYC and EGFR, which were closely related to drug resistance, and there was mutual regulation between ecDNA molecules derived from these genes (56). Therefore, ecDNA can make cancer cells acquire drug resistance through high expression of drug resistance-related genes.

### 3.3 eccDNA is integrated into chromosome to form HSR

The amplification of oncogenes or drug-resistant genes, which plays a pivotal role in human cell malignant transformation, confers a growth advantage to the cells through the overproduction of the amplified gene product. In cytogenetic research, the amplified gene is located in ecDNA or HSR (57).

HSR was initially detected in Chinese hamster cells resistant to MTX in 1976. Moreover, HSR are longer segments of chromosome than any single band in the karyotype. The staining intensity of HSRs in G-band staining was medium, rather than the normal pattern of alternating dark and light bands in the rest of the chromosome (58). ecDNA and HSR could be converted to each other and are homologous sequences (59). The structure of ecDNA is unstable. ecDNA can be integrated into the chromosome arm, where it efficiently initiated the breakage-fusion-bridge cycle (BFB) that generated HSR (10). As described above, the ABCG2 gene carried by ecDNA in GBM (19) and the DHFR gene carried by ecDNA in HeLa cells resistant to MTX (44) were integrated into a certain site of the chromosome to form HSR and produce a stable condition, so that the drug resistance of cancer cells was more stable. There is evidence that, compared with oncogenes or drug resistance genes amplified on ecDNA, HSR-amplified oncogenes or drug resistance genes located in chromosomes are not easy to eliminate from cells (60).

### 3.4 Dynamic regulation of gene expression by eccDNA

Previously, it was reported that EGFR derived from adult GBM was often mutated to produce a constitutively active oncogenic variant, EGFRvIII (18, 61). EGFRvIII amplification on ecDNA can provide growth advantages for cancer cells and make cancer cells more sensitive to TKI treatment (34, 62). After stopping TKI treatment, the amount of ecDNA carrying EGFRvIII may increase again, suggesting that ecDNA deletion of EGFRvIII leads to TKI resistance, allowing cancer to adapt to its growth environment and evade treatment against oncogenes maintained on ecDNA (18). This suggests a highly specific dynamic mechanism, in contrast to cancer cells amplifying resistance genes through ecDNA to improve drug resistance, highlighting the diversity and complexity of ecDNA-promoting resistance mechanisms.

## 4 Clinical application prospect for eccDNA

**Figure 3:**
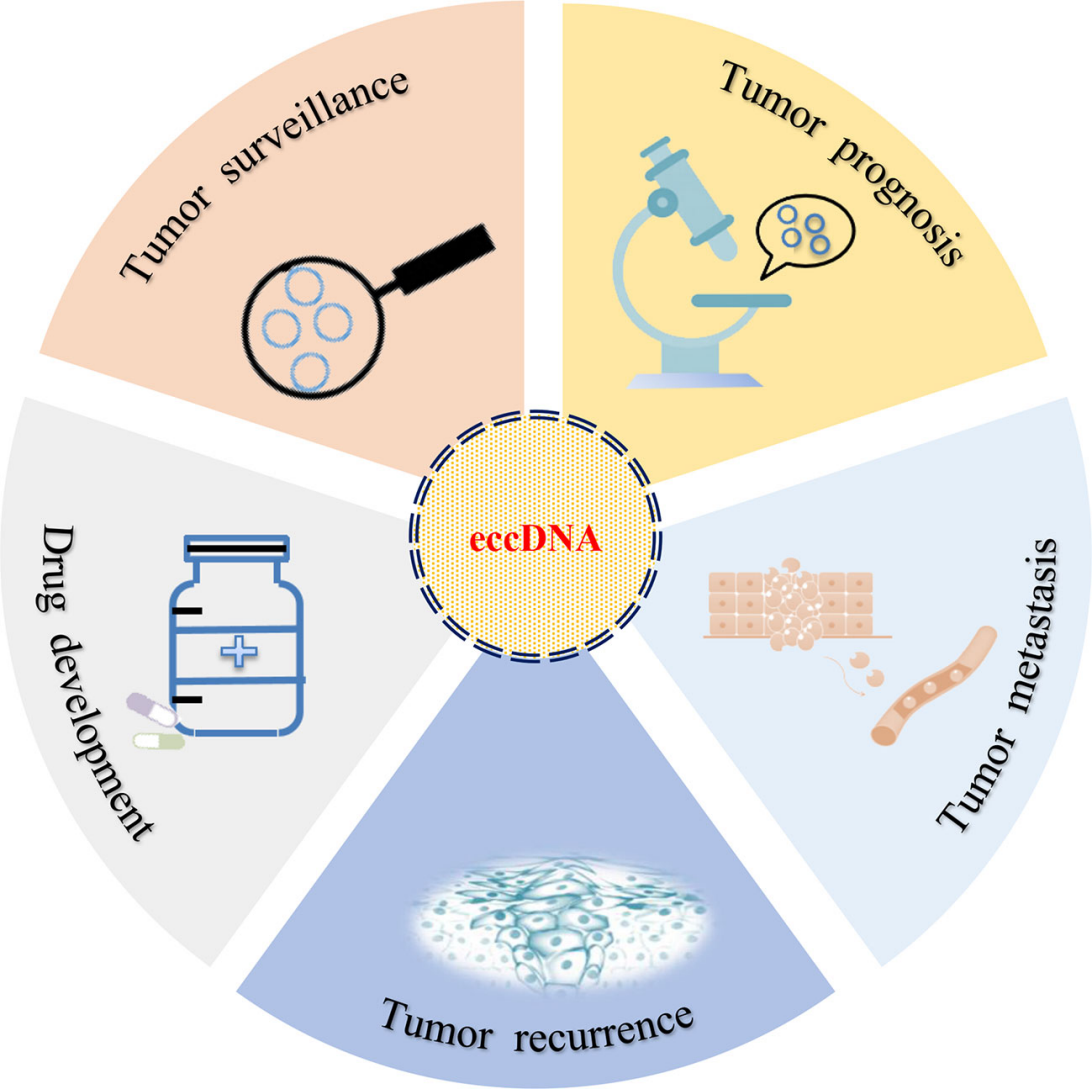
Clinical implications of eccDNA. eccDNA may be used as a biomarker for tumor surveillance, prognosis, metastasis, and recurrence. In addition, the development of target drugs may also provide a new strategy for the treatment of cancer in clinical setting.

The relationship between eccDNA and cancer has been studied for decades. With the development of next-generation sequencing (NGS) and the completion of human genome sequencing, the clinical application of eccDNA became an area of intense research interest (**Figure 3**). However, in the same patients, studies had identified that the average length of human ovarian cancer eccDNA was slightly longer than that of normal tissue, and the circulating eccDNA of patients after tumor resection was usually shorter than before operation (63). This phenomenon has also been confirmed in lung cancer (63). In acute myeloid leukemia (AML), the amount of eccDNA in cancer cells far exceeds that in normal cells. The average number of eccDNA gradually increased as primitive cells differentiated into terminal cells, and multiple recurrent and specific eccDNA were also identified in abnormal and normal cells (64). If the enrichment of eccDNA, especially the change of sequence, can predict the development of benign diseases into malignant cancers (such as hepatitis into liver cancer), eccDNA should be exhibited as a promising biomarker for cancer monitoring and prognosis.

Amplification of oncogenes and drug resistance genes in ecDNA promotes tumorigenesis and drug resistance. Many studies in recent years have confirmed that ecDNA is highly opened and contains enhancer sequences. These characteristics have improved its transcriptional activity (53, 54). These findings revealed a new understanding of eccDNA. Furthermore, when used as an immune stimulus, the circular nature of eccDNA can endow immune cells, such as dendritic cells and macrophages, with super immune capacity. The level of cytokines induced by eccDNA is far higher than that of linear DNA, which will help us better understand the pathogenesis of some inflammation-related tumors (65). As a result, combining gene therapy with immunotherapy can improve the efficacy of traditional anticancer drug therapy while also providing a new idea for the development of new anticancer drugs.

Kristen et al. performed whole-genome sequencing on 17 different cancer types, revealing that the frequency of ecDNA varies with cancer type (66). This phenomenon has almost never been found in normal cells, indicating that eccDNA in the blood of cancer patients may be used as a promising tool to track and determine the source and type of cancer. Although there are few studies on the role of eccDNA in tumor recurrence at present, a study on neuroblastoma demonstrated that cancer cells with an invasive phenotype of DM might be the source of tumor recurrence, which largely depends on the internal heterogeneity of tumors (67). The latest study by Cen et al. also reported the important role of eccDNA in tumor metastasis. They explored the eccDNA profile in high-grade serous ovarian cancer (HGSOC) by circle-sequencing analysis and found that the expression of DNMT1^circle10302690-10302961^ (identified a novel eccDNA) was significantly downregulated in metastatic HGSOC tumor tissues and its reduction was associated with poor prognosis in HGSOC patients (68). Moreover, eccDNA can express functional small regulatory RNAs, including microRNA (miRNA). These miRNAs would regulate the downstream signal pathway (12), such as miR-145 (69), miR-191 (70), and miR-126 (71), by promoting tumor angiogenesis through related kinase signal transduction and transcriptional activation. In addition, the coding of oncogenes by eccDNA has been widely confirmed, such as c-myc (cellular-myelocytomatosis viral oncogene). C-myc promoted the expression of S100A4 (S100 Calcium Binding Protein A4) in prostate cancer cells by affecting downstream signaling molecules, which played an important role in tumor metastasis (72). Another example is EGFR, which promotes the invasion and metastasis of GBM by regulating the expression of matrix metalloproteinase-9 (MMP-9) (73). However, the exact mechanism of eccDNA directly mediating tumor metastasis remains to be studied.

## 5 Conclusion

EccDNA is widely found in various tumor tissues. Its unique genetic characteristics allow the number of oncogenes or drug-resistance genes in cells to increase sharply, resulting in a higher level of gene expression in cancer cells and providing tumor heterogeneity, which will contribute to cancer progression and resistance to chemotherapy. In addition, the diversity of somatic mutations in human cancer genomes also promoted the evolution of eccDNA. Some scholars attributed these mutations to the activity of the APOBEC3 (Apolipoprotein B mRNA Editing Catalytic Polypeptide-like) enzyme, which is a cytosine deaminase in cells. APOBEC3 can treat circular ecDNA as foreign viruses and try to limit or cut them. In this process, APOBEC3 induces the formation of mutation clusters within a single ecDNA molecule, which in turn plays a key role in accelerating cancer evolution and possibly leading to drug resistance (74). Here, we propose the following prospects regarding the regulation of eccDNA in drug resistance: (1) The particular molecular mechanism of resistance mediated by eccDNA in cancers needs to be investigated further. (2) According to the synthesis of microDNA mimics and its transcription *in vitro* and *in vivo*, microDNA can be transcribed into the functional, small regulatory microRNAs, which can regulate the expression of drug resistance genes (12). This synthesis might be a potential method to investigate the relationship between eccDNA and the regulation of gene expression. (3) The interactions between the multiple drug resistance genes on eccDNA and multitarget drugs can be deeply explored. (4) Most studies of eccDNA in tumor drug resistance are currently limited to the cell and animal level, and further population study should be conducted to improve the clinical significance of eccDNA as a biomarker. In a word, research on many scientific issues about eccDNA has revealed a new mechanism of cancer progression and regulation of chemotherapy resistance. Targeting specific genes and regulatory elements of eccDNA will hopefully become a therapeutic strategy for clinical cancer treatment.

## Author contributions

JL contributed to the data collection and manuscript writing. YL contributed to the data collection and manuscript writing. TZ contributed to the manuscript revision. TX contributed to the manuscript revision. CC contributed to the manuscript revision. ML contributed to the manuscript revision. QQ contributed to the manuscript revision. YS contributed to the study design and manuscript revision. SW contributed to the study design and manuscript writing and revision. All authors contributed to the article and approved the submitted version.

## References

[1] Xia C Dong X Li H Cao M Sun D He S . Cancer statistics in China and united states, 2022: Profiles, trends, and determinants. Chin Med J (2022) 135(5):584–90. doi: 10.1097/cm9.0000000000002108 PMC8920425 35143424

[2] Pérez-Herrero E Fernández-Medarde A . Advanced targeted therapies in cancer: Drug nanocarriers, the future of chemotherapy. Eur J pharm biopharm (2015) 93:52–79. doi: 10.1016/j.ejpb.2015.03.018 25813885

[3] Nikolaou M Pavlopoulou A Georgakilas AG Kyrodimos E . The challenge of drug resistance in cancer treatment: A current overview. Clin Exp metastasis (2018) 35(4):309–18. doi: 10.1007/s10585-018-9903-0 29799080

[4] Lippert TH Ruoff HJ Volm M . Intrinsic and acquired drug resistance in malignant tumors. the main reason for therapeutic failure. Arzneimittel-Forschung (2008) 58(6):261–4. doi: 10.1055/s-0031-1296504 18677966

[5] Chatterjee N Bivona TG . Polytherapy and targeted cancer drug resistance. Trends Cancer (2019) 5(3):170–82. doi: 10.1016/j.trecan.2019.02.003 PMC6446041 30898264

[6] Konieczkowski DJ Johannessen CM Garraway LA . A convergence-based framework for cancer drug resistance. Cancer Cell (2018) 33(5):801–15. doi: 10.1016/j.ccell.2018.03.025 PMC5957297 29763622

[7] Shoshani O Brunner SF Yaeger R Ly P Nechemia-Arbely Y Kim DH . Chromothripsis drives the evolution of gene amplification in cancer. Nature (2021) 591(7848):137–41. doi: 10.1038/s41586-020-03064-z PMC7933129 33361815

[8] Wu S Bafna V Chang HY Mischel PS . Extrachromosomal DNA: An emerging hallmark in human cancer. Annu Rev Pathol (2022) 17:367–86. doi: 10.1146/annurev-pathmechdis-051821-114223 PMC9125980 34752712

[9] Liao Z Jiang W Ye L Li T Yu X Liu L . Classification of extrachromosomal circular DNA with a focus on the role of extrachromosomal DNA (Ecdna) in tumor heterogeneity and progression. Biochim Biophys Acta Rev Cancer (2020) 1874(1):188392. doi: 10.1016/j.bbcan.2020.188392 32735964

[10] Shimizu N . Gene amplification and the extrachromosomal circular DNA. Genes (2021) 12(10):1533. doi: 10.3390/genes12101533 34680928 PMC8535887

[11] Wang T Zhang H Zhou Y Shi J . Extrachromosomal circular DNA: A new potential role in cancer progression. J Trans Med (2021) 19(1):257. doi: 10.1186/s12967-021-02927-x PMC8194206 34112178

[12] Paulsen T Shibata Y Kumar P Dillon L Dutta A . Small extrachromosomal circular dnas, microdna, produce short regulatory rnas that suppress gene expression independent of canonical promoters. Nucleic Acids Res (2019) 47(9):4586–96. doi: 10.1093/nar/gkz155 PMC6511871 30828735

[13] Mehanna P Gagné V Lajoie M Spinella JF St-Onge P Sinnett D . Characterization of the microdna through the response to chemotherapeutics in lymphoblastoid cell lines. PloS One (2017) 12(9):e0184365. doi: 10.1371/journal.pone.0184365 28877255 PMC5587290

[14] Cohen S Regev A Lavi S . Small polydispersed circular DNA (Spcdna) in human cells: Association with genomic instability. Oncogene (1997) 14(8):977–85. doi: 10.1038/sj.onc.1200917 9050997

[15] Tomaska L Nosek J Kramara J Griffith JD . Telomeric circles: Universal players in telomere maintenance? Nat Struct Mol Biol (2009) 16(10):1010–5. doi: 10.1038/nsmb.1660 PMC4041010 19809492

[16] Tomaska L McEachern MJ Nosek J . Alternatives to telomerase: Keeping linear chromosomes *Via* telomeric circles. FEBS Lett (2004) 567(1):142–6. doi: 10.1016/j.febslet.2004.04.058 15165907

[17] Wu P Liu Y Zhou R Liu L Zeng H Xiong F . Extrachromosomal circular DNA: A new target in cancer. Front Oncol (2022) 12:814504. doi: 10.3389/fonc.2022.814504 35494014 PMC9046939

[18] Nathanson DA Gini B Mottahedeh J Visnyei K Koga T Gomez G . Targeted therapy resistance mediated by dynamic regulation of extrachromosomal mutant egfr DNA. Sci (New York NY) (2014) 343(6166):72–6. doi: 10.1126/science.1241328 PMC4049335 24310612

[19] Rao VK Wangsa D Robey RW Huff L Honjo Y Hung J . Characterization of Abcg2 gene amplification manifesting as extrachromosomal DNA in mitoxantrone-selected Sf295 human glioblastoma cells. Cancer Genet Cytogen (2005) 160(2):126–33. doi: 10.1016/j.cancergencyto.2004.12.013 15993268

[20] Valent A Bénard J Clausse B Barrois M Valteau-Couanet D Terrier-Lacombe MJ . *In vivo* elimination of acentric double minutes containing amplified mycn from neuroblastoma tumor cells through the formation of micronuclei. Am J Pathol (2001) 158(5):1579–84. doi: 10.1016/s0002-9440(10)64112-0 PMC1891958 11337354

[21] Ruiz-Herrera A Smirnova A Khoriauli L Nergadze SG Mondello C Giulotto E . Gene amplification in human cells knocked down for Rad54. Genome Integrity (2011) 2(1):5. doi: 10.1186/2041-9414-2-5 21418575 PMC3074559

[22] Meng X Qi X Guo H Cai M Li C Zhu J . Novel role for non-homologous end joining in the formation of double minutes in methotrexate-resistant colon cancer cells. J Med Genet (2015) 52(2):135–44. doi: 10.1136/jmedgenet-2014-102703 PMC4316941 25537274

[23] Ruiz JC Choi KH von Hoff DD Roninson IB Wahl GM . Autonomously replicating episomes contain Mdr1 genes in a multidrug-resistant human cell line. Mol Cell Biol (1989) 9(1):109–15. doi: 10.1128/mcb.9.1.109-115.1989 PMC362151 2648129

[24] Schoenlein PV Shen DW Barrett JT Pastan I Gottesman MM . Double minute chromosomes carrying the human multidrug resistance 1 and 2 genes are generated from the dimerization of submicroscopic circular dnas in colchicine-selected kb carcinoma cells. Mol Biol Cell (1992) 3(5):507–20. doi: 10.1091/mbc.3.5.507 PMC275604 1611154

[25] Lin C Chen Y Zhang F Liu B Xie C Song Y . Encoding gene Rab3b exists in linear chromosomal and circular extrachromosomal DNA and contributes to cisplatin resistance of hypopharyngeal squamous cell carcinoma *Via* inducing autophagy. Cell Death Dis (2022) 13(2):171. doi: 10.1038/s41419-022-04627-w 35194030 PMC8863882

[26] Von Hoff DD Waddelow T Forseth B Davidson K Scott J Wahl G . Hydroxyurea accelerates loss of extrachromosomally amplified genes from tumor cells. Cancer Res (1991) 51(23 Pt 1):6273–9. 1682044

[27] Curt GA Carney DN Cowan KH Jolivet J Bailey BD Drake JC . Unstable methotrexate resistance in human small-cell carcinoma associated with double minute chromosomes. New Engl J Med (1983) 308(4):199–202. doi: 10.1056/nejm198301273080406 6294518

[28] Sakai K Wake N Fujino T Yasuda T Kato H Fujimoto S . Methotrexate-resistant mechanisms in human choriocarcinoma cells. Gynecol Oncol (1989) 34(1):7–11. doi: 10.1016/0090-8258(89)90095-4 2737530

[29] Hahn P Nevaldine B Morgan WF . X-Ray induction of methotrexate resistance due to dhfr gene amplification. Somatic Cell Mol Genet (1990) 16(5):413–23. doi: 10.1007/bf01233191 2122527

[30] Møller HD Lin L Xiang X Petersen TS Huang J Yang L . Crispr-c: Circularization of genes and chromosome by crispr in human cells. Nucleic Acids Res (2018) 46(22):e131. doi: 10.1093/nar/gky767 30551175 PMC6294522

[31] Lange JT Rose JC Chen CY Pichugin Y Xie L Tang J . The evolutionary dynamics of extrachromosomal DNA in human cancers. Nat Genet (2022) 54(10):1527–33. doi: 10.1038/s41588-022-01177-x PMC9534767 36123406

[32] Polivka J Jr. Polivka J Holubec L Kubikova T Priban V Hes O . Advances in experimental targeted therapy and immunotherapy for patients with glioblastoma multiforme. Anticancer Res (2017) 37(1):21–33. doi: 10.21873/anticanres.11285 28011470

[33] Snuderl M Fazlollahi L Le LP Nitta M Zhelyazkova BH Davidson CJ . Mosaic amplification of multiple receptor tyrosine kinase genes in glioblastoma. Cancer Cell (2011) 20(6):810–7. doi: 10.1016/j.ccr.2011.11.005 22137795

[34] Vivanco I Robins HI Rohle D Campos C Grommes C Nghiemphu PL . Differential sensitivity of glioma- versus lung cancer-specific egfr mutations to egfr kinase inhibitors. Cancer Discov (2012) 2(5):458–71. doi: 10.1158/2159-8290.Cd-11-0284 PMC3354723 22588883

[35] Stec WJ Rosiak K Siejka P Peciak J Popeda M Banaszczyk M . Cell line with endogenous egfrviii expression is a suitable model for research and drug development purposes. Oncotarget (2016) 7(22):31907–25. doi: 10.18632/oncotarget.8201 PMC5077985 27004406

[36] Kukal S Guin D Rawat C Bora S Mishra MK Sharma P . Multidrug efflux transporter Abcg2: Expression and regulation. Cell Mol Life Sci CMLS (2021) 78(21-22):6887–939. doi: 10.1007/s00018-021-03901-y PMC11072723 34586444

[37] Zafar A Wang W Liu G Wang X Xian W McKeon F . Molecular targeting therapies for neuroblastoma: Progress and challenges. Med Res Rev (2021) 41(2):961–1021. doi: 10.1002/med.21750 33155698 PMC7906923

[38] Nicolai S Pieraccioli M Peschiaroli A Melino G Raschellà G . Neuroblastoma: Oncogenic mechanisms and therapeutic exploitation of necroptosis. Cell Death Dis (2015) 6(12):e2010. doi: 10.1038/cddis.2015.354 26633716 PMC4720889

[39] Zaman GJ Flens MJ van Leusden MR de Haas M Mülder HS Lankelma J . The human multidrug resistance-associated protein mrp is a plasma membrane drug-efflux pump. Proc Natl Acad Sci USA (1994) 91(19):8822–6. doi: 10.1073/pnas.91.19.8822 PMC44698 7916458

[40] Hong J Jing S Zhang Y Chen R Owusu-Ansah KG Chen B . Y-320, a novel immune-modulator, sensitizes multidrug-resistant tumors to chemotherapy. Am J Trans Res (2020) 12(2):551–62. PMC7061851 32194903

[41] Canute GW Longo SL Longo JA Winfield JA Nevaldine BH Hahn PJ . Hydroxyurea accelerates the loss of epidermal growth factor receptor genes amplified as double-minute chromosomes in human glioblastoma multiforme. Neurosurgery (1996) 39(5):976–83. doi: 10.1097/00006123-199611000-00019 8905754

[42] Van den Berg C Von Hoff DD . Use of hydroxyurea to alter drug resistance of human tumor cells. Cancer Treat Res (1995) 78:95–114. doi: 10.1007/978-1-4615-2007-8_5 8595149

[43] Hu Z Ma D . The precision prevention and therapy of hpv-related cervical cancer: New concepts and clinical implications. Cancer Med (2018) 7(10):5217–36. doi: 10.1002/cam4.1501 PMC6198240 30589505

[44] Singer MJ Mesner LD Friedman CL Trask BJ Hamlin JL . Amplification of the human dihydrofolate reductase gene *Via* double minutes is initiated by chromosome breaks. Proc Natl Acad Sci USA (2000) 97(14):7921–6. doi: 10.1073/pnas.130194897 PMC16646 10859355

[45] Morales C García MJ Ribas M Miró R Muñoz M Caldas C . Dihydrofolate reductase amplification and sensitization to methotrexate of methotrexate-resistant colon cancer cells. Mol Cancer Ther (2009) 8(2):424–32. doi: 10.1158/1535-7163.Mct-08-0759 19190117

[46] Cai M Zhang H Hou L Gao W Song Y Cui X . Inhibiting homologous recombination decreases extrachromosomal amplification but has no effect on intrachromosomal amplification in methotrexate-resistant colon cancer cells. Int J Cancer (2019) 144(5):1037–48. doi: 10.1002/ijc.31781 PMC6586039 30070702

[47] Sanchez AM Barrett JT Schoenlein PV . Fractionated ionizing radiation accelerates loss of amplified Mdr1 genes harbored by extrachromosomal DNA in tumor cells. Cancer Res (1998) 58(17):3845–54. 9731494

[48] Newman JR Connolly TM Illing EA Kilgore ML Locher JL Carroll WR . Survival trends in hypopharyngeal cancer: A population-based review. Laryngosc (2015) 125(3):624–9. doi: 10.1002/lary.24915 25220657

[49] Arai A Chano T Futami K Furuichi Y Ikebuchi K Inui T . Recql1 and wrn proteins are potential therapeutic targets in head and neck squamous cell carcinoma. Cancer Res (2011) 71(13):4598–607. doi: 10.1158/0008-5472.Can-11-0320 21571861

[50] Zhao XG Sun RJ Yang XY Liu DY Lei DP Jin T . Chloroquine-enhanced efficacy of cisplatin in the treatment of hypopharyngeal carcinoma in xenograft mice. PloS One (2015) 10(4):e0126147. doi: 10.1371/journal.pone.0126147 25923669 PMC4414471

[51] Parmigiani G Boca S Lin J Kinzler KW Velculescu V Vogelstein B . Design and analysis issues in genome-wide somatic mutation studies of cancer. Genomics (2009) 93(1):17–21. doi: 10.1016/j.ygeno.2008.07.005 18692126 PMC2820387

[52] Marusyk A Polyak K . Tumor heterogeneity: Causes and consequences. Biochim Biophys Acta (2010) 1805(1):105–17. doi: 10.1016/j.bbcan.2009.11.002 PMC2814927 19931353

[53] Wu S Turner KM Nguyen N Raviram R Erb M Santini J . Circular ecdna promotes accessible chromatin and high oncogene expression. Nature (2019) 575(7784):699–703. doi: 10.1038/s41586-019-1763-5 31748743 PMC7094777

[54] Hung KL Yost KE Xie L Shi Q Helmsauer K Luebeck J . Ecdna hubs drive cooperative intermolecular oncogene expression. Nature (2021) 600(7890):731–6. doi: 10.1038/s41586-021-04116-8 PMC9126690 34819668

[55] Morton AR Dogan-Artun N Faber ZJ MacLeod G Bartels CF Piazza MS . Functional enhancers shape extrachromosomal oncogene amplifications. Cell (2019) 179(6):1330–41.e13. doi: 10.1016/j.cell.2019.10.039 31761532 PMC7241652

[56] Zhu Y Gujar AD Wong CH Tjong H Ngan CY Gong L . Oncogenic extrachromosomal DNA functions as mobile enhancers to globally amplify chromosomal transcription. Cancer Cell (2021) 39(5):694–707.e7. doi: 10.1016/j.ccell.2021.03.006 33836152 PMC8119378

[57] Cowell JK . Double minutes and homogeneously staining regions: Gene amplification in mammalian cells. Annu Rev Genet (1982) 16:21–59. doi: 10.1146/annurev.ge.16.120182.000321 6760799

[58] Biedler JL Spengler BA . Metaphase chromosome anomaly: Association with drug resistance and cell-specific products. Sci (New York NY) (1976) 191(4223):185–7. doi: 10.1126/science.942798 942798

[59] Von Hoff DD Forseth B Clare CN Hansen KL VanDevanter D . Double minutes arise from circular extrachromosomal DNA intermediates which integrate into chromosomal sites in human hl-60 leukemia cells. J Clin Invest (1990) 85(6):1887–95. doi: 10.1172/jci114650 PMC296655 2189894

[60] Benner SE Wahl GM Von Hoff DD . Double minute chromosomes and homogeneously staining regions in tumors taken directly from patients versus in human tumor cell lines. Anti-cancer Drugs (1991) 2(1):11–25. doi: 10.1097/00001813-199102000-00002 1720337

[61] Garraway LA Jänne PA . Circumventing cancer drug resistance in the era of personalized medicine. Cancer Discov (2012) 2(3):214–26. doi: 10.1158/2159-8290.Cd-12-0012 22585993

[62] Inda MM Bonavia R Mukasa A Narita Y Sah DW Vandenberg S . Tumor heterogeneity is an active process maintained by a mutant egfr-induced cytokine circuit in glioblastoma. Genes Dev (2010) 24(16):1731–45. doi: 10.1101/gad.1890510 PMC2922502 20713517

[63] Kumar P Dillon LW Shibata Y Jazaeri AA Jones DR Dutta A . Normal and cancerous tissues release extrachromosomal circular DNA (Eccdna) into the circulation. Mol Cancer Res (2017) 15(9):1197–205. doi: 10.1158/1541-7786.Mcr-17-0095 PMC5581709 28550083

[64] Zeng T Huang W Cui L Zhu P Lin Q Zhang W . The landscape of extrachromosomal circular DNA (Eccdna) in the normal hematopoiesis and leukemia evolution. Cell Death Discov (2022) 8(1):400. doi: 10.1038/s41420-022-01189-w 36171187 PMC9519993

[65] Wang Y Wang M Djekidel MN Chen H Liu D Alt FW . Eccdnas are apoptotic products with high innate immunostimulatory activity. Nature (2021) 599(7884):308–14. doi: 10.1038/s41586-021-04009-w PMC9295135 34671165

[66] Turner KM Deshpande V Beyter D Koga T Rusert J Lee C . Extrachromosomal oncogene amplification drives tumour evolution and genetic heterogeneity. Nature (2017) 543(7643):122–5. doi: 10.1038/nature21356 PMC5334176 28178237

[67] Pajic M Norris MD Cohn SL Haber M . The role of the multidrug resistance-associated protein 1 gene in neuroblastoma biology and clinical outcome. Cancer Lett (2005) 228(1-2):241–6. doi: 10.1016/j.canlet.2005.01.060 15979785

[68] Cen Y Fang Y Ren Y Hong S Lu W Xu J . Global characterization of extrachromosomal circular dnas in advanced high grade serous ovarian cancer. Cell Death Dis (2022) 13(4):342. doi: 10.1038/s41419-022-04807-8 35418185 PMC9007969

[69] Ye D Shen Z Zhou S . Function of microrna-145 and mechanisms underlying its role in malignant tumor diagnosis and treatment. Cancer Manage Res (2019) 11:969–79. doi: 10.2147/cmar.S191696 PMC6349084 30774425

[70] Xu W Luo F Sun B Ye H Li J Shi L . Hif-2α, acting *Via* mir-191, is involved in angiogenesis and metastasis of arsenite-transformed hbe cells. Toxicol Res (2016) 5(1):66–78. doi: 10.1039/c5tx00225g PMC6060623 30090327

[71] Meister J Schmidt MHH . Mir-126 and mir-126*: New players in cancer. TheScientificWorldJournal (2010) 10:2090–100. doi: 10.1100/tsw.2010.198 PMC5763667 20953557

[72] Amatangelo MD Goodyear S Varma D Stearns ME . C-myc expression and Mek1-induced Erk2 nuclear localization are required for tgf-beta induced epithelial-mesenchymal transition and invasion in prostate cancer. Carcinogenesis (2012) 33(10):1965–75. doi: 10.1093/carcin/bgs227 PMC3463154 22791812

[73] Zhou YH Chen Y Hu Y Yu L Tran K Giedzinski E . The role of egfr double minutes in modulating the response of malignant gliomas to radiotherapy. Oncotarget (2017) 8(46):80853–68. doi: 10.18632/oncotarget.20714 PMC5655244 29113349

[74] Bergstrom EN Luebeck J Petljak M Khandekar A Barnes M Zhang T . Mapping clustered mutations in cancer reveals Apobec3 mutagenesis of ecdna. Nature (2022) 602(7897):510–7. doi: 10.1038/s41586-022-04398-6 PMC8850194 35140399

